# Bis(1-ferrocenylethanone oximato)triphenyl­anti­mony(V)

**DOI:** 10.1107/S1600536808039834

**Published:** 2008-12-06

**Authors:** Jinshi Fan

**Affiliations:** a College of Chemical Engineering, Qingdao University of Science and Technology, Qingdao 266042, People’s Republic of China

## Abstract

In the title compound, [Fe_2_Sb(C_5_H_5_)_2_(C_6_H_5_)_3_(C_7_H_7_NO)_2_] or [Sb(C_6_H_5_)_3_{Fe(C_5_H_5_)(C_7_H_7_NO)}_2_], the Sb center has a slightly distorted trigonal-bipyramidal geometry, with the three phenyl ligands in equatorial positions and the two O atoms from the ferrocenylethanone oximate ligands in axial positions. The crystal structure is stabilized by two inter­molecular C—H⋯π inter­actions.

## Related literature

For anti­mony compounds with cytotoxicity and anti­tumor activities, see: Takahashi *et al.* (2002 ▶). For a related structure, see: Sharma *et al.* (2003 ▶).
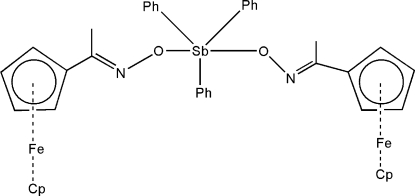


## Experimental

### 

#### Crystal data

[[Fe_2_Sb(C_5_H_5_)_2_(C_6_H_5_)_3_(C_7_H_7_NO)_2_]
*M* *_r_* = 837.20
Orthorhombic, 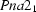

*a* = 19.921 (2) Å
*b* = 19.938 (2) Å
*c* = 9.371 (1) Å
*V* = 3722.0 (7) Å^3^
*Z* = 4
Mo *K*α radiation
μ = 1.53 mm^−1^
*T* = 298 (2) K
0.42 × 0.36 × 0.11 mm

#### Data collection

Bruker SMART CCD diffractometer
Absorption correction: multi-scan (*SADABS*; Sheldrick, 1996 ▶) *T*_min_ = 0.566, *T*_max_ = 0.850
15019 measured reflections
6305 independent reflections
4319 reflections with *I* > 2σ(*I*)
*R*_int_ = 0.054

#### Refinement

*R*[*F* ^2^ > 2σ(*F* ^2^)] = 0.050
*wR*(*F* ^2^) = 0.119
*S* = 0.95
6305 reflections
442 parameters
1 restraint
H-atom parameters constrained
Δρ_max_ = 0.66 e Å^−3^
Δρ_min_ = −0.38 e Å^−3^
Absolute structure: Flack (1983 ▶), 2803 Friedel pairs
Flack parameter: −0.03 (3)

### 

Data collection: *SMART* (Bruker 1998 ▶); cell refinement: *SAINT* (Bruker 1998 ▶); data reduction: *SAINT*; program(s) used to solve structure: *SHELXS97* (Sheldrick, 2008 ▶); program(s) used to refine structure: *SHELXL97* (Sheldrick, 2008 ▶); molecular graphics: *SHELXTL* (Sheldrick, 2008 ▶) and *DIAMOND* (Brandenburg, 1998 ▶); software used to prepare material for publication: *SHELXL97*.

## Supplementary Material

Crystal structure: contains datablocks I, global. DOI: 10.1107/S1600536808039834/lx2068sup1.cif

Structure factors: contains datablocks I. DOI: 10.1107/S1600536808039834/lx2068Isup2.hkl

Additional supplementary materials:  crystallographic information; 3D view; checkCIF report

## Figures and Tables

**Table 1:** Hydrogen-bond geometry (Å, °)

*D*—H⋯*A*	*D*—H	H⋯*A*	*D*⋯*A*	*D*—H⋯*A*
C11—H11⋯*Cg*1^i^	0.93	2.78	3.677 (4)	163
C21—H21⋯*Cg*2^ii^	0.93	3.03	3.751 (3)	136

Symmetry codes: (i) 
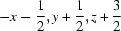
; (ii) 
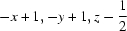
. *Cg*1 and *Cg*2 are the centroids of the C15–C19 cyclo­penta­dienyl ring and the C25–C30 benzene ring, respectively.

**Fig. 1.**
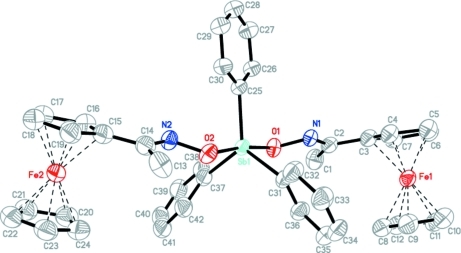
The molecular structure of (I) with displacement ellipsoids for non-hydrogen atoms drawn at the 30% probability level.

**Fig. 2.**
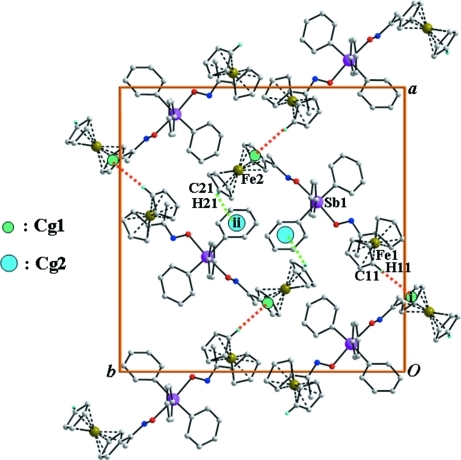
C—H···π interactions (dotted lines) in the title compound. Cg denotes the ring centroid. [Symmetry code: (i) x - 1/2, -y + 1/2, z + 1; (ii) -x + 1, -y + 1, z - 1/2.]

## supplementary crystallographic information

### Comment

Research on some main group and early transition metal complexes with internally functionalized oximes have shown that oximes were an important class of N/O donor ligands having different coordinating abilities with the metal centers (Sharma, *et al.*, 2003). On the other hand, antimony compounds have been reported with good cytotoxicity and antitumor activities, some of them can affect the repair of the DNA-double strand break (Takahashi *et al.*, 2002). However, to our best knowledge, corresponding triorganoantimony (V) compounds with these ligands were hitherto unknown. Here we report the crystal structure of the title compound, bis(acetylferrocenyoximato)triphenylantimony(V) (Fig. 1).

The compound was an interesting heterometallic (Sb, Fe) compound (Fig.1). The Sb atom is five-coordinated with a distorted trigonal-bipyramidal geometry (Table 1, Fig.1). Around the central Sb atom, atoms C25, C31, C37 occupy the equatorial plane, while O1 and O2 lie in axial sites. The axial bond angle O2—Sb1—O1 [173.5 (2)°] deviates from linearity by 6.5°. The sum of C31—Sb1—C37 [118.1 (4)°], C31—Sb1—C25 [122.3 (4)°] and C37—Sb1—C25 [119.6 (4)°] bond angles is 360°, which shows that these atoms have slightly deviations from ideal trigonal-bipyramidal geometry. The crystal structure is stabilized by two intermolecular C—H···π interactions (Table 1 and Fig. 2); one between a cyclopentadienyl-H atom and the cyclopentadienyl ring of a neighbouring molecule, with a C11—H11···*Cg*1^i^ separation of 2.78 Å, a second between a cyclopentadienyl H atom and the benzene ring of an adjacent molecule, with a C21—H21···*Cg*2^ii^ separation of 3.03 Å (*Cg*1 and *Cg*2 are the centroids of the C15–C19 cyclopentadienyl ring and the C25–C30 benzene ring, respectively, symmetry code as in Fig. 2).

### Experimental

Acetylferrocenyloxime (1.46 g, 6 mmol) was added to a stirring solution containing dibromotriphenylantimony (1.54 g, 3 mmol) in tetrahydrofuran (50 ml). After stirring for 12 h at room temperature the orange solution was obtained and then filtered. The resulting clear solution was evaporated under vacuum until the orange solid is obtained. The solid was recrystallized from ethanol to give orange crystals, yield 72%, decomposition temperature 485 K. Anal. Calcd (%) for C_42_H_39_Fe_2_N_2_O_2_Sb: C, 60.25; H, 4.70; N, 3.35%; Found: C, 60.96; H, 4.83; N, 3.72%.

### Refinement

H atoms were positioned geometrically [0.93 (CH), and 0.96 (CH_3_) Å] and constrained to ride on their parent atoms with *U*_iso_(H) = 1.2(1.5 for methyl)*U*_eq_.

### Crystal data

**Table:**

[Fe_2_Sb(C_5_H_5_)_2_(C_6_H_5_)_3_(C_7_H_7_NO)_2_]	*F*(000) = 1696
*M**_r_* = 837.20	*D*_x_ = 1.494 Mg m^−^^3^
Orthorhombic, *P**n**a*2_1_	Mo *K*α radiation, λ = 0.71073 Å
Hall symbol: P 2c -2n	Cell parameters from 3650 reflections
*a* = 19.921 (2) Å	θ = 2.6–20.9°
*b* = 19.938 (2) Å	µ = 1.53 mm^−^^1^
*c* = 9.371 (1) Å	*T* = 298 K
*V* = 3722.0 (7) Å^3^	Block, orange
*Z* = 4	0.42 × 0.36 × 0.11 mm

### Data collection

**Table:**

Bruker SMART CCD diffractometer	6305 independent reflections
Radiation source: fine-focus sealed tube	4319 reflections with *I* > 2σ(*I*)
graphite	*R*_int_ = 0.054
Detector resolution: 10.0 pixels mm^-1^	θ_max_ = 25.0°, θ_min_ = 1.4°
φ and ω scans	*h* = −23→22
Absorption correction: multi-scan (*SADABS*; Sheldrick, 1996)	*k* = −16→23
*T*_min_ = 0.566, *T*_max_ = 0.850	*l* = −11→11
15019 measured reflections	

### Refinement

**Table:**

Refinement on *F*^2^	Secondary atom site location: difference Fourier map
Least-squares matrix: full	Hydrogen site location: difference Fourier map
*R*[*F*^2^ > 2σ(*F*^2^)] = 0.050	H-atom parameters constrained
*wR*(*F*^2^) = 0.119	*w* = 1/[σ^2^(*F*_o_^2^) + (0.0603*P*)^2^] where *P* = (*F*_o_^2^ + 2*F*_c_^2^)/3
*S* = 0.95	(Δ/σ)_max_ < 0.001
6305 reflections	Δρ_max_ = 0.66 e Å^−^^3^
442 parameters	Δρ_min_ = −0.38 e Å^−^^3^
1 restraint	Absolute structure: Flack (1983), 2803 Friedel pairs
Primary atom site location: structure-invariant direct methods	Flack parameter: −0.03 (3)

### Special details

**Table:**

Geometry. All e.s.d.'s (except the e.s.d. in the dihedral angle between two l.s. planes) are estimated using the full covariance matrix. The cell e.s.d.'s are taken into account individually in the estimation of e.s.d.'s in distances, angles and torsion angles; correlations between e.s.d.'s in cell parameters are only used when they are defined by crystal symmetry. An approximate (isotropic) treatment of cell e.s.d.'s is used for estimating e.s.d.'s involving l.s. planes.
Refinement. Refinement of *F*^2^ against ALL reflections. The weighted *R*-factor *wR* and goodness of fit *S* are based on *F*^2^, conventional *R*-factors *R* are based on *F*, with *F* set to zero for negative *F*^2^. The threshold expression of *F*^2^ > σ(*F*^2^) is used only for calculating *R*-factors(gt) *etc*. and is not relevant to the choice of reflections for refinement. *R*-factors based on *F*^2^ are statistically about twice as large as those based on *F*, and *R*- factors based on ALL data will be even larger.

### Fractional atomic coordinates and isotropic or equivalent isotropic displacement parameters (Å^2^)

**Table:**

	*x*	*y*	*z*	*U*_iso_*/*U*_eq_	
Sb1	0.59602 (2)	0.30774 (2)	0.45296 (7)	0.05361 (15)	
Fe1	0.45127 (7)	0.10703 (6)	0.89653 (14)	0.0681 (4)	
Fe2	0.70848 (6)	0.58368 (6)	0.14551 (14)	0.0652 (4)	
N1	0.5302 (4)	0.1883 (4)	0.5553 (8)	0.0640 (19)	
N2	0.6814 (4)	0.4088 (3)	0.3258 (7)	0.0633 (18)	
O1	0.5168 (2)	0.2409 (2)	0.4595 (8)	0.0659 (13)	
O2	0.6689 (2)	0.3812 (3)	0.4621 (8)	0.0670 (13)	
C1	0.4100 (5)	0.1668 (5)	0.5137 (12)	0.090 (3)	
H1A	0.4043	0.2139	0.4960	0.134*	
H1B	0.3753	0.1515	0.5767	0.134*	
H1C	0.4074	0.1428	0.4251	0.134*	
C2	0.4784 (6)	0.1548 (5)	0.5823 (11)	0.078 (3)	
C3	0.4860 (6)	0.1007 (5)	0.6887 (12)	0.085 (3)	
C4	0.5387 (6)	0.0953 (5)	0.7878 (13)	0.091 (3)	
H4	0.5753	0.1240	0.7954	0.110*	
C5	0.5255 (6)	0.0374 (6)	0.8757 (12)	0.097 (3)	
H5	0.5538	0.0214	0.9470	0.116*	
C6	0.4659 (7)	0.0095 (6)	0.8392 (12)	0.094 (4)	
H6	0.4467	−0.0281	0.8814	0.113*	
C7	0.4370 (6)	0.0479 (5)	0.7241 (13)	0.093 (3)	
H7	0.3956	0.0407	0.6807	0.112*	
C8	0.4162 (7)	0.2018 (6)	0.9327 (18)	0.096 (4)	
H8	0.4195	0.2383	0.8715	0.116*	
C9	0.4622 (7)	0.1856 (6)	1.0319 (14)	0.100 (4)	
H9	0.5014	0.2096	1.0493	0.120*	
C10	0.4431 (8)	0.1279 (7)	1.1052 (14)	0.108 (4)	
H10	0.4667	0.1043	1.1745	0.129*	
C11	0.3801 (7)	0.1145 (6)	1.0489 (15)	0.104 (4)	
H11	0.3515	0.0812	1.0826	0.125*	
C12	0.3649 (6)	0.1579 (7)	0.9337 (17)	0.108 (4)	
H12	0.3280	0.1565	0.8730	0.130*	
C13	0.7584 (5)	0.4761 (5)	0.4745 (13)	0.096 (3)	
H13A	0.7239	0.4852	0.5434	0.144*	
H13B	0.7854	0.5155	0.4611	0.144*	
H13C	0.7861	0.4400	0.5079	0.144*	
C14	0.7265 (5)	0.4567 (5)	0.3346 (11)	0.077 (3)	
C15	0.7438 (5)	0.4917 (5)	0.2045 (14)	0.082 (3)	
C16	0.7102 (6)	0.4871 (5)	0.0709 (14)	0.091 (3)	
H16	0.6735	0.4597	0.0515	0.109*	
C17	0.7422 (6)	0.5317 (5)	−0.0299 (16)	0.095 (3)	
H17	0.7298	0.5399	−0.1241	0.114*	
C18	0.7966 (6)	0.5604 (6)	0.0468 (14)	0.095 (3)	
H18	0.8280	0.5897	0.0077	0.114*	
C19	0.7969 (5)	0.5387 (5)	0.1889 (13)	0.089 (3)	
H19	0.8265	0.5525	0.2600	0.107*	
C20	0.6112 (6)	0.6074 (7)	0.1771 (16)	0.101 (4)	
H20	0.5758	0.5776	0.1638	0.121*	
C21	0.6387 (6)	0.6498 (6)	0.0766 (14)	0.099 (4)	
H21	0.6235	0.6548	−0.0166	0.119*	
C22	0.6925 (6)	0.6836 (5)	0.1362 (15)	0.095 (3)	
H22	0.7203	0.7144	0.0909	0.114*	
C23	0.6966 (6)	0.6626 (6)	0.2767 (16)	0.098 (3)	
H23	0.7283	0.6764	0.3433	0.118*	
C24	0.6461 (6)	0.6181 (6)	0.3000 (15)	0.096 (3)	
H24	0.6368	0.5979	0.3872	0.115*	
C25	0.6560 (5)	0.2430 (4)	0.3274 (9)	0.067 (2)	
C26	0.6275 (6)	0.1856 (5)	0.2682 (11)	0.079 (3)	
H26	0.5828	0.1745	0.2849	0.095*	
C27	0.6682 (6)	0.1454 (5)	0.1832 (11)	0.089 (3)	
H27	0.6500	0.1074	0.1406	0.107*	
C28	0.7336 (6)	0.1602 (6)	0.1611 (12)	0.091 (3)	
H28	0.7600	0.1315	0.1066	0.109*	
C29	0.7608 (6)	0.2159 (6)	0.2169 (12)	0.087 (3)	
H29	0.8054	0.2262	0.1976	0.104*	
C30	0.7232 (5)	0.2582 (5)	0.3030 (11)	0.080 (3)	
H30	0.7425	0.2961	0.3437	0.096*	
C31	0.6042 (5)	0.3120 (4)	0.6763 (10)	0.065 (2)	
C32	0.6653 (6)	0.3155 (5)	0.7442 (11)	0.085 (3)	
H32	0.7049	0.3126	0.6923	0.102*	
C33	0.6669 (6)	0.3238 (5)	0.8957 (12)	0.092 (3)	
H33	0.7079	0.3241	0.9431	0.110*	
C34	0.6108 (6)	0.3311 (5)	0.9689 (15)	0.082 (3)	
H34	0.6122	0.3368	1.0673	0.099*	
C35	0.5531 (6)	0.3302 (5)	0.9021 (12)	0.082 (3)	
H35	0.5138	0.3361	0.9541	0.099*	
C36	0.5492 (5)	0.3210 (4)	0.7595 (11)	0.078 (3)	
H36	0.5071	0.3207	0.7166	0.094*	
C37	0.5264 (5)	0.3719 (5)	0.3527 (11)	0.074 (3)	
C38	0.4780 (5)	0.3472 (6)	0.2615 (12)	0.083 (3)	
H38	0.4764	0.3016	0.2405	0.100*	
C39	0.4309 (6)	0.3916 (7)	0.2003 (13)	0.095 (4)	
H39	0.3996	0.3756	0.1350	0.115*	
C40	0.4311 (6)	0.4554 (7)	0.2353 (14)	0.094 (4)	
H40	0.3994	0.4839	0.1946	0.112*	
C41	0.4770 (6)	0.4817 (6)	0.3310 (14)	0.095 (4)	
H41	0.4750	0.5265	0.3581	0.114*	
C42	0.5258 (5)	0.4398 (5)	0.3852 (12)	0.088 (3)	
H42	0.5589	0.4574	0.4444	0.106*	

### Atomic displacement parameters (Å^2^)

**Table:**

	*U*^11^	*U*^22^	*U*^33^	*U*^12^	*U*^13^	*U*^23^
Sb1	0.0594 (3)	0.0579 (3)	0.0435 (3)	−0.0023 (3)	0.0035 (3)	0.0021 (3)
Fe1	0.0767 (9)	0.0633 (7)	0.0644 (8)	−0.0096 (7)	0.0159 (7)	0.0021 (6)
Fe2	0.0649 (8)	0.0635 (7)	0.0673 (9)	0.0028 (6)	0.0009 (7)	0.0003 (6)
N1	0.073 (5)	0.064 (5)	0.055 (5)	0.000 (4)	0.013 (4)	0.009 (4)
N2	0.072 (5)	0.065 (4)	0.053 (4)	−0.013 (4)	−0.001 (4)	0.002 (4)
O1	0.067 (3)	0.070 (3)	0.061 (3)	−0.011 (3)	0.001 (4)	0.015 (4)
O2	0.067 (3)	0.081 (3)	0.053 (3)	−0.014 (3)	0.003 (4)	−0.003 (4)
C1	0.089 (8)	0.089 (7)	0.091 (8)	−0.025 (6)	0.000 (6)	0.008 (6)
C2	0.095 (8)	0.069 (6)	0.070 (7)	−0.001 (6)	0.027 (6)	0.000 (5)
C3	0.102 (8)	0.077 (7)	0.075 (8)	0.000 (6)	0.028 (7)	0.003 (6)
C4	0.101 (8)	0.089 (8)	0.084 (8)	0.006 (7)	0.029 (7)	0.012 (6)
C5	0.111 (9)	0.092 (8)	0.088 (8)	0.007 (7)	0.025 (8)	0.013 (7)
C6	0.113 (10)	0.087 (8)	0.083 (9)	−0.004 (7)	0.031 (7)	0.004 (6)
C7	0.109 (8)	0.085 (7)	0.084 (8)	−0.007 (7)	0.024 (7)	−0.001 (6)
C8	0.112 (9)	0.091 (8)	0.086 (12)	0.006 (7)	0.025 (8)	−0.002 (7)
C9	0.117 (10)	0.094 (9)	0.091 (9)	0.000 (8)	0.014 (8)	−0.004 (7)
C10	0.126 (12)	0.110 (10)	0.088 (10)	0.005 (9)	0.020 (9)	0.003 (8)
C11	0.112 (10)	0.101 (9)	0.098 (10)	−0.011 (8)	0.044 (9)	−0.001 (8)
C12	0.112 (9)	0.116 (9)	0.098 (12)	0.008 (8)	0.022 (9)	−0.008 (9)
C13	0.106 (8)	0.075 (6)	0.107 (10)	−0.006 (6)	−0.031 (8)	0.006 (6)
C14	0.075 (7)	0.068 (6)	0.087 (8)	0.003 (6)	0.005 (6)	0.000 (5)
C15	0.079 (7)	0.073 (7)	0.093 (9)	0.009 (6)	0.009 (7)	0.003 (6)
C16	0.095 (8)	0.084 (8)	0.094 (9)	0.006 (6)	0.013 (7)	−0.003 (6)
C17	0.103 (8)	0.085 (6)	0.097 (8)	0.009 (6)	0.012 (9)	−0.005 (8)
C18	0.094 (9)	0.085 (8)	0.105 (10)	0.009 (7)	0.018 (8)	0.004 (7)
C19	0.085 (8)	0.080 (7)	0.102 (10)	0.005 (6)	0.003 (7)	0.004 (6)
C20	0.095 (9)	0.096 (9)	0.111 (11)	0.013 (7)	0.005 (8)	−0.003 (8)
C21	0.103 (9)	0.093 (8)	0.101 (10)	0.017 (8)	0.000 (8)	0.000 (8)
C22	0.103 (10)	0.083 (8)	0.098 (10)	0.011 (7)	0.002 (8)	0.001 (7)
C23	0.106 (10)	0.084 (7)	0.104 (11)	0.019 (7)	0.003 (8)	−0.005 (7)
C24	0.098 (9)	0.093 (8)	0.097 (10)	0.018 (7)	0.013 (8)	−0.001 (7)
C25	0.078 (7)	0.069 (6)	0.054 (5)	0.015 (5)	0.015 (5)	0.004 (5)
C26	0.098 (7)	0.077 (7)	0.063 (6)	0.019 (6)	0.020 (6)	0.001 (5)
C27	0.105 (9)	0.086 (7)	0.075 (8)	0.011 (7)	0.018 (7)	−0.007 (6)
C28	0.105 (9)	0.091 (8)	0.078 (8)	0.020 (7)	0.025 (7)	0.001 (7)
C29	0.092 (8)	0.088 (7)	0.080 (8)	0.017 (7)	0.026 (6)	0.002 (6)
C30	0.091 (8)	0.079 (7)	0.069 (7)	0.021 (6)	0.015 (6)	0.001 (5)
C31	0.071 (6)	0.076 (6)	0.049 (5)	0.002 (5)	0.004 (5)	0.008 (4)
C32	0.086 (7)	0.108 (8)	0.061 (7)	0.006 (6)	0.004 (6)	0.001 (6)
C33	0.096 (8)	0.112 (8)	0.067 (7)	0.005 (7)	−0.012 (7)	0.001 (6)
C34	0.095 (8)	0.099 (7)	0.054 (7)	−0.010 (6)	0.007 (7)	−0.001 (6)
C35	0.097 (8)	0.089 (7)	0.060 (7)	0.006 (6)	0.010 (6)	0.000 (5)
C36	0.087 (7)	0.093 (7)	0.054 (6)	0.012 (6)	0.000 (6)	−0.001 (5)
C37	0.071 (7)	0.080 (7)	0.069 (7)	0.014 (5)	0.014 (6)	0.017 (5)
C38	0.078 (7)	0.095 (7)	0.076 (8)	0.020 (6)	0.012 (6)	0.023 (6)
C39	0.083 (8)	0.114 (10)	0.090 (9)	0.011 (8)	0.010 (7)	0.026 (7)
C40	0.083 (8)	0.105 (10)	0.092 (9)	0.023 (7)	0.013 (7)	0.035 (7)
C41	0.090 (8)	0.097 (8)	0.099 (9)	0.022 (7)	0.018 (7)	0.025 (7)
C42	0.080 (7)	0.094 (8)	0.091 (7)	0.017 (6)	0.009 (6)	0.025 (6)

### Geometric parameters (Å, °)

**Table:**

Sb1—O2	2.064 (5)	C13—H13B	0.9600
Sb1—O1	2.067 (5)	C13—H13C	0.9600
Sb1—C31	2.101 (9)	C14—C15	1.447 (14)
Sb1—C37	2.107 (10)	C15—C19	1.421 (13)
Sb1—C25	2.117 (9)	C15—C16	1.423 (15)
Fe1—C10	2.006 (13)	C16—C17	1.445 (15)
Fe1—C11	2.018 (11)	C16—H16	0.9300
Fe1—C7	2.021 (11)	C17—C18	1.422 (15)
Fe1—C9	2.027 (12)	C17—H17	0.9300
Fe1—C12	2.028 (11)	C18—C19	1.400 (15)
Fe1—C4	2.031 (10)	C18—H18	0.9300
Fe1—C6	2.038 (11)	C19—H19	0.9300
Fe1—C5	2.038 (11)	C20—C24	1.362 (16)
Fe1—C8	2.042 (11)	C20—C21	1.379 (15)
Fe1—C3	2.070 (10)	C20—H20	0.9300
Fe2—C23	2.010 (12)	C21—C22	1.384 (14)
Fe2—C20	2.018 (12)	C21—H21	0.9300
Fe2—C19	2.019 (11)	C22—C23	1.385 (16)
Fe2—C22	2.020 (10)	C22—H22	0.9300
Fe2—C21	2.022 (11)	C23—C24	1.358 (14)
Fe2—C24	2.027 (12)	C23—H23	0.9300
Fe2—C18	2.038 (11)	C24—H24	0.9300
Fe2—C15	2.041 (10)	C25—C30	1.390 (12)
Fe2—C16	2.049 (11)	C25—C26	1.393 (12)
Fe2—C17	2.056 (12)	C26—C27	1.390 (13)
N1—C2	1.255 (11)	C26—H26	0.9300
N1—O1	1.406 (9)	C27—C28	1.352 (14)
N2—C14	1.312 (11)	C27—H27	0.9300
N2—O2	1.414 (9)	C28—C29	1.341 (14)
C1—C2	1.525 (15)	C28—H28	0.9300
C1—H1A	0.9600	C29—C30	1.388 (13)
C1—H1B	0.9600	C29—H29	0.9300
C1—H1C	0.9600	C30—H30	0.9300
C2—C3	1.477 (14)	C31—C36	1.356 (12)
C3—C4	1.405 (15)	C31—C32	1.374 (13)
C3—C7	1.474 (13)	C32—C33	1.429 (14)
C4—C5	1.443 (14)	C32—H32	0.9300
C4—H4	0.9300	C33—C34	1.319 (14)
C5—C6	1.355 (14)	C33—H33	0.9300
C5—H5	0.9300	C34—C35	1.309 (14)
C6—C7	1.442 (15)	C34—H34	0.9300
C6—H6	0.9300	C35—C36	1.351 (13)
C7—H7	0.9300	C35—H35	0.9300
C8—C9	1.345 (18)	C36—H36	0.9300
C8—C12	1.346 (15)	C37—C38	1.380 (13)
C8—H8	0.9300	C37—C42	1.389 (14)
C9—C10	1.391 (16)	C38—C39	1.412 (14)
C9—H9	0.9300	C38—H38	0.9300
C10—C11	1.387 (17)	C39—C40	1.315 (15)
C10—H10	0.9300	C39—H39	0.9300
C11—C12	1.417 (17)	C40—C41	1.383 (15)
C11—H11	0.9300	C40—H40	0.9300
C12—H12	0.9300	C41—C42	1.378 (13)
C13—C14	1.507 (14)	C41—H41	0.9300
C13—H13A	0.9600	C42—H42	0.9300
			
O2—Sb1—O1	173.5 (2)	Fe1—C8—H8	126.8
O2—Sb1—C31	82.8 (3)	C8—C9—C10	110.7 (13)
O1—Sb1—C31	93.2 (3)	C8—C9—Fe1	71.3 (8)
O2—Sb1—C37	92.9 (3)	C10—C9—Fe1	69.0 (7)
O1—Sb1—C37	84.4 (3)	C8—C9—H9	124.7
C31—Sb1—C37	118.1 (4)	C10—C9—H9	124.7
O2—Sb1—C25	93.4 (3)	Fe1—C9—H9	126.7
O1—Sb1—C25	93.1 (3)	C11—C10—C9	102.7 (13)
C31—Sb1—C25	122.3 (4)	C11—C10—Fe1	70.3 (8)
C37—Sb1—C25	119.6 (4)	C9—C10—Fe1	70.6 (8)
C10—Fe1—C11	40.3 (5)	C11—C10—H10	128.7
C10—Fe1—C7	152.8 (5)	C9—C10—H10	128.7
C11—Fe1—C7	120.7 (5)	Fe1—C10—H10	122.3
C10—Fe1—C9	40.4 (5)	C10—C11—C12	111.4 (12)
C11—Fe1—C9	64.9 (5)	C10—C11—Fe1	69.4 (7)
C7—Fe1—C9	165.1 (5)	C12—C11—Fe1	69.9 (7)
C10—Fe1—C12	70.1 (6)	C10—C11—H11	124.3
C11—Fe1—C12	41.0 (5)	C12—C11—H11	124.3
C7—Fe1—C12	108.1 (5)	Fe1—C11—H11	128.2
C9—Fe1—C12	66.2 (5)	C8—C12—C11	103.9 (13)
C10—Fe1—C4	125.6 (6)	C8—C12—Fe1	71.2 (7)
C11—Fe1—C4	165.0 (6)	C11—C12—Fe1	69.1 (7)
C7—Fe1—C4	69.7 (5)	C8—C12—H12	128.1
C9—Fe1—C4	108.1 (5)	C11—C12—H12	128.1
C12—Fe1—C4	150.6 (5)	Fe1—C12—H12	123.4
C10—Fe1—C6	117.8 (5)	C14—C13—H13A	109.5
C11—Fe1—C6	110.9 (5)	C14—C13—H13B	109.5
C7—Fe1—C6	41.6 (4)	H13A—C13—H13B	109.5
C9—Fe1—C6	152.4 (6)	C14—C13—H13C	109.5
C12—Fe1—C6	130.1 (5)	H13A—C13—H13C	109.5
C4—Fe1—C6	68.6 (4)	H13B—C13—H13C	109.5
C10—Fe1—C5	107.1 (5)	N2—C14—C15	117.4 (10)
C11—Fe1—C5	128.9 (5)	N2—C14—C13	122.0 (9)
C7—Fe1—C5	68.2 (5)	C15—C14—C13	120.5 (9)
C9—Fe1—C5	120.6 (6)	C19—C15—C16	107.6 (10)
C12—Fe1—C5	166.7 (5)	C19—C15—C14	125.7 (12)
C4—Fe1—C5	41.6 (4)	C16—C15—C14	126.7 (11)
C6—Fe1—C5	38.8 (4)	C19—C15—Fe2	68.7 (6)
C10—Fe1—C8	67.6 (6)	C16—C15—Fe2	69.9 (6)
C11—Fe1—C8	64.8 (5)	C14—C15—Fe2	125.4 (7)
C7—Fe1—C8	128.6 (6)	C15—C16—C17	109.2 (11)
C9—Fe1—C8	38.6 (5)	C15—C16—Fe2	69.3 (6)
C12—Fe1—C8	38.6 (4)	C17—C16—Fe2	69.7 (6)
C4—Fe1—C8	118.9 (4)	C15—C16—H16	125.4
C6—Fe1—C8	167.2 (6)	C17—C16—H16	125.4
C5—Fe1—C8	153.4 (5)	Fe2—C16—H16	127.2
C10—Fe1—C3	163.1 (6)	C18—C17—C16	104.6 (12)
C11—Fe1—C3	154.8 (6)	C18—C17—Fe2	69.0 (7)
C7—Fe1—C3	42.2 (4)	C16—C17—Fe2	69.1 (7)
C9—Fe1—C3	126.9 (5)	C18—C17—H17	127.7
C12—Fe1—C3	118.4 (6)	C16—C17—H17	127.7
C4—Fe1—C3	40.0 (4)	Fe2—C17—H17	125.7
C6—Fe1—C3	69.3 (4)	C19—C18—C17	111.1 (11)
C5—Fe1—C3	68.0 (4)	C19—C18—Fe2	69.1 (7)
C8—Fe1—C3	109.1 (5)	C17—C18—Fe2	70.3 (7)
C23—Fe2—C20	67.3 (5)	C19—C18—H18	124.4
C23—Fe2—C19	109.1 (5)	C17—C18—H18	124.4
C20—Fe2—C19	156.0 (6)	Fe2—C18—H18	127.8
C23—Fe2—C22	40.2 (5)	C18—C19—C15	107.3 (11)
C20—Fe2—C22	67.9 (5)	C18—C19—Fe2	70.6 (7)
C19—Fe2—C22	125.8 (5)	C15—C19—Fe2	70.3 (6)
C23—Fe2—C21	66.7 (5)	C18—C19—H19	126.3
C20—Fe2—C21	39.9 (4)	C15—C19—H19	126.3
C19—Fe2—C21	162.6 (5)	Fe2—C19—H19	124.4
C22—Fe2—C21	40.1 (4)	C24—C20—C21	106.2 (13)
C23—Fe2—C24	39.3 (4)	C24—C20—Fe2	70.7 (7)
C20—Fe2—C24	39.4 (4)	C21—C20—Fe2	70.2 (7)
C19—Fe2—C24	122.8 (5)	C24—C20—H20	126.9
C22—Fe2—C24	66.4 (5)	C21—C20—H20	126.9
C21—Fe2—C24	65.5 (5)	Fe2—C20—H20	123.9
C23—Fe2—C18	123.9 (5)	C20—C21—C22	109.3 (12)
C20—Fe2—C18	161.4 (6)	C20—C21—Fe2	69.9 (7)
C19—Fe2—C18	40.4 (4)	C22—C21—Fe2	69.9 (7)
C22—Fe2—C18	110.0 (5)	C20—C21—H21	125.3
C21—Fe2—C18	126.6 (5)	C22—C21—H21	125.3
C24—Fe2—C18	158.3 (6)	Fe2—C21—H21	126.4
C23—Fe2—C15	125.3 (5)	C21—C22—C23	106.3 (12)
C20—Fe2—C15	120.2 (5)	C21—C22—Fe2	70.0 (7)
C19—Fe2—C15	41.0 (4)	C23—C22—Fe2	69.5 (7)
C22—Fe2—C15	162.3 (5)	C21—C22—H22	126.8
C21—Fe2—C15	155.5 (5)	C23—C22—H22	126.8
C24—Fe2—C15	108.8 (5)	Fe2—C22—H22	125.2
C18—Fe2—C15	67.7 (4)	C24—C23—C22	107.9 (13)
C23—Fe2—C16	161.1 (5)	C24—C23—Fe2	71.0 (7)
C20—Fe2—C16	106.7 (5)	C22—C23—Fe2	70.3 (7)
C19—Fe2—C16	68.7 (5)	C24—C23—H23	126.1
C22—Fe2—C16	156.2 (6)	C22—C23—H23	126.1
C21—Fe2—C16	121.0 (5)	Fe2—C23—H23	124.2
C24—Fe2—C16	124.9 (5)	C23—C24—C20	110.2 (13)
C18—Fe2—C16	67.4 (5)	C23—C24—Fe2	69.7 (7)
C15—Fe2—C16	40.7 (4)	C20—C24—Fe2	69.9 (7)
C23—Fe2—C17	157.2 (5)	C23—C24—H24	124.9
C20—Fe2—C17	123.3 (5)	C20—C24—H24	124.9
C19—Fe2—C17	69.7 (5)	Fe2—C24—H24	127.1
C22—Fe2—C17	120.9 (5)	C30—C25—C26	120.5 (9)
C21—Fe2—C17	107.3 (5)	C30—C25—Sb1	120.1 (7)
C24—Fe2—C17	160.3 (5)	C26—C25—Sb1	119.5 (7)
C18—Fe2—C17	40.6 (4)	C27—C26—C25	117.6 (10)
C15—Fe2—C17	69.6 (5)	C27—C26—H26	121.2
C16—Fe2—C17	41.2 (4)	C25—C26—H26	121.2
C2—N1—O1	111.6 (8)	C28—C27—C26	121.6 (11)
C14—N2—O2	110.3 (7)	C28—C27—H27	119.2
N1—O1—Sb1	110.7 (5)	C26—C27—H27	119.2
N2—O2—Sb1	111.3 (5)	C29—C28—C27	120.7 (11)
C2—C1—H1A	109.5	C29—C28—H28	119.7
C2—C1—H1B	109.5	C27—C28—H28	119.7
H1A—C1—H1B	109.5	C28—C29—C30	120.8 (11)
C2—C1—H1C	109.5	C28—C29—H29	119.6
H1A—C1—H1C	109.5	C30—C29—H29	119.6
H1B—C1—H1C	109.5	C29—C30—C25	118.8 (10)
N1—C2—C3	116.1 (11)	C29—C30—H30	120.6
N1—C2—C1	124.4 (10)	C25—C30—H30	120.6
C3—C2—C1	119.4 (10)	C36—C31—C32	116.2 (10)
C4—C3—C7	107.0 (9)	C36—C31—Sb1	121.0 (7)
C4—C3—C2	125.3 (10)	C32—C31—Sb1	122.2 (7)
C7—C3—C2	127.3 (11)	C31—C32—C33	119.1 (10)
C4—C3—Fe1	68.4 (6)	C31—C32—H32	120.5
C7—C3—Fe1	67.1 (6)	C33—C32—H32	120.5
C2—C3—Fe1	123.8 (7)	C34—C33—C32	120.6 (11)
C3—C4—C5	107.6 (10)	C34—C33—H33	119.7
C3—C4—Fe1	71.5 (6)	C32—C33—H33	119.7
C5—C4—Fe1	69.5 (6)	C35—C34—C33	119.6 (13)
C3—C4—H4	126.2	C35—C34—H34	120.2
C5—C4—H4	126.2	C33—C34—H34	120.2
Fe1—C4—H4	124.4	C34—C35—C36	121.7 (11)
C6—C5—C4	110.1 (12)	C34—C35—H35	119.2
C6—C5—Fe1	70.6 (7)	C36—C35—H35	119.2
C4—C5—Fe1	68.9 (6)	C35—C36—C31	122.7 (11)
C6—C5—H5	125.0	C35—C36—H36	118.7
C4—C5—H5	125.0	C31—C36—H36	118.7
Fe1—C5—H5	127.1	C38—C37—C42	118.6 (10)
C5—C6—C7	108.8 (11)	C38—C37—Sb1	121.3 (8)
C5—C6—Fe1	70.6 (7)	C42—C37—Sb1	120.0 (9)
C7—C6—Fe1	68.6 (6)	C37—C38—C39	119.5 (11)
C5—C6—H6	125.6	C37—C38—H38	120.3
C7—C6—H6	125.6	C39—C38—H38	120.3
Fe1—C6—H6	126.8	C40—C39—C38	120.2 (13)
C6—C7—C3	106.4 (11)	C40—C39—H39	119.9
C6—C7—Fe1	69.8 (7)	C38—C39—H39	119.9
C3—C7—Fe1	70.7 (6)	C39—C40—C41	122.0 (12)
C6—C7—H7	126.8	C39—C40—H40	119.0
C3—C7—H7	126.8	C41—C40—H40	119.0
Fe1—C7—H7	124.3	C42—C41—C40	118.5 (11)
C9—C8—C12	110.9 (13)	C42—C41—H41	120.8
C9—C8—Fe1	70.1 (7)	C40—C41—H41	120.8
C12—C8—Fe1	70.2 (7)	C41—C42—C37	121.0 (11)
C9—C8—H8	124.5	C41—C42—H42	119.5
C12—C8—H8	124.5	C37—C42—H42	119.5

### Hydrogen-bond geometry (Å, °)

**Table:**

*D*—H···*A*	*D*—H	H···*A*	*D*···*A*	*D*—H···*A*
C11—H11···Cg1^i^	0.93	2.78	3.677 (4)	163
C21—H21···Cg2^ii^	0.93	3.03	3.751 (3)	136

Symmetry codes: (i) −*x*−1/2, *y*+1/2, *z*+3/2; (ii) −*x*+1, −*y*+1, *z*−1/2.
